# Metabolic Characteristics of Frail Older People with Diabetes Mellitus—A Systematic Search for Phenotypes

**DOI:** 10.3390/metabo13060705

**Published:** 2023-05-29

**Authors:** Ahmed H. Abdelhafiz, Grace L. Keegan, Alan J. Sinclair

**Affiliations:** 1 Department of Geriatric Medicine, Rotherham General Hospital, Moorgate Road, Rotherham S60 2UD, UK; 2 Foundation for Diabetes Research in Older People, Diabetes Frail Ltd., Droitwich Spa WR9 0QH, UK; 3 King’s College, London WC2R 2LS, UK

**Keywords:** older people, frailty, diabetes mellitus, metabolic, phenotypes

## Abstract

Frailty in older people with diabetes is viewed as one homogeneous category. We previously suggested that frailty is not homogeneous and spans across a metabolic spectrum that starts with an anorexic malnourished (AM) frail phenotype and ends with a sarcopenic obese (SO) phenotype. We aimed to investigate the metabolic characteristics of frail older people with diabetes reported in the current literature to explore whether they fit into two distinctive metabolic phenotypes. We performed systematic review of studies published over the last 10 years and reported characteristics of frail older people with diabetes mellitus. A total of 25 studies were included in this systematic review. Fifteen studies reported frail patients’ characteristics that could fit into an AM phenotype. This phenotype is characterised by low body weight, increased prevalence of malnutrition markers such as low serum albumin, low serum cholesterol, low Hb, low HbA1c, and increased risk of hypoglycaemia. Ten studies reported frail patients’ characteristics that describe a SO phenotype. This phenotype is characterised by increased body weight, increased serum cholesterol, high HbA1c, and increased blood glucose levels. Due to significant weight loss in the AM phenotype, insulin resistance decreases, leading to a decelerated diabetes trajectory and reduced hypoglycaemic agent use or deintensification of therapy. On the other hand, in the SO phenotype, insulin resistance increases leading to accelerated diabetes trajectory and increased hypoglycaemic agent use or intensification of therapy. Current literature suggests that frailty is a metabolically heterogeneous condition that includes AM and SO phenotypes. Both phenotypes have metabolically distinctive features, which will have a different effect on diabetes trajectory. Therefore, clinical decision-making and future clinical studies should consider the metabolic heterogeneity of frailty.

## 1. Introduction

The current global prevalence of diabetes mellitus is around 10.5% in adults aged 20–79 years and is expected to increase by 46% in the year 2045 [1]. The prevalence is increasing with increasing age due to increased life expectancy and it peaks at 24% in older people aged between 75–79 years [1]. In addition to the traditional diabetes-related cardiovascular disease, frailty emerges as a new complication category affecting about 32–48% of older people with diabetes [2,3,4,5]. As a result, frailty is now increasingly recognised by clinical guidelines, although these guidelines view frail older people as one homogenous group [6,7]. The clinical guidelines, generally, recommend relaxed glycaemic targets for frail older people with diabetes due to their physical dysfunction and their increased risk of hypoglycaemia [6,7]. However, frail older people are likely to be metabolically heterogeneous and have a range of different metabolic profiles [8]. Although frailty is associated with weight loss, which is not a mandatory requisite for frailty diagnosis, frail individuals can also suffer from obesity [9,10]. In addition, frailty is associated with muscle mass loss or sarcopenia [9,11]. Muscle mass loss in frailty is mainly affecting the insulin-resistant type II muscle fibres more than the insulin-sensitive type I fibres, which may reduce the overall insulin resistance [12,13]. Therefore, in frail individuals, the overall insulin sensitivity of the individual will be determined by the net combination of the differential muscle fibres loss, total muscle mass, amount of visceral fat accumulation, and the severity of weight loss. This may result in a range of metabolic profiles with varying degrees of insulin sensitivity among frail older people with diabetes. Therefore, frailty is likely to have a metabolic spectrum, which may present as an anorexic malnourished (AM) individual with significant weight loss at one end and at the other extreme with the sarcopenic obese (SO) individual due to a significant increase in visceral fat and loss of muscle mass [8]. In between these two phenotypes, there will be individuals with varying degrees of fat/muscle ratios and corresponding varying degrees of insulin sensitivities leading to a few intermediate phenotypes. The AM phenotype, due to significant weight loss and reduced insulin resistance, are likely to have lower HbA1c, markers of malnutrition, frequent hypoglycaemia, and the need for less hypoglycaemic therapy. On the other hand, the SO phenotype, due to weight gain and increased insulin resistance, will have higher HbA1c, markers of metabolic syndrome due to visceral obesity, and the use of more hypoglycaemic therapy. We previously referred to the metabolic phenotypes of frailty and its impact on clinical decision-making regarding glycaemic control, the burden of medications, the choice of hypoglycaemic agents and overall goals of therapy [8]. We also suggested that there will be a need for future studies to further explore the metabolic heterogeneity of frailty. Due to the difficulty in recruiting and conducting research in frail older people, we searched the existing literature to identify and group together the metabolic characteristics of frail older people with diabetes to explore whether they fit into the two hypothesised metabolic profiles. 

## 2. Aims

We aimed to investigate the metabolic and anthropometric characteristics of frail older people with diabetes reported in the current literature to explore whether they can fit into two distinctive metabolic phenotypes. 

## 3. Materials and Methods

### 3.1. Data Source

**Figure 1:**
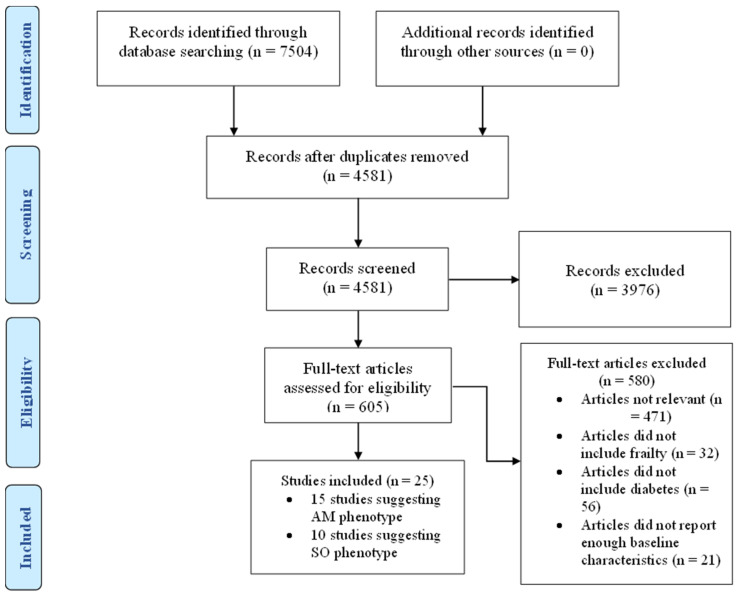
PRISMA flow diagram. AM = Anorexic malnourished, SO = Sarcopenic obese.

In this systematic review, we undertook a detailed search of the literature for studies that reported clinical characteristics of frail patients with diabetes in accordance with the Preferred Reporting Items for Systematic Reviews and Meta-Analyses (PRISMA) recommendations. (Figure 1) A full assessment of relevant articles was conducted by searching the following databases: Medline, EMBASE, and CINHAL. We used Medical Subject Heading (MeSH) terms including frailty, frail, older people, elderly, and diabetes mellitus. We reviewed all the articles initially by titles and abstract then full text. Within the text, we then searched for glycaemic control, HbA1c, outcomes, risk factors, physical function, cognitive function, hypoglycaemia, hyperglycaemia, mortality, quality of life, health care utilisation, hospitalisation, care home admission, adverse events, body weight, body mass index, muscle mass, muscle mass index, waist circumference, metabolic, characteristics, baseline, criteria, predictors, as individual words and combined phrases. The research strategy is available in Supplementary Materials. We also performed a manual search of the citations in the relevant articles to retrieve further related studies. We searched Medline for articles published only in the English language over the last 10 years from 1 January 2013 to 31 December 2022. 

### 3.2. Study Selection

Studies were included if they reported all or some of the baseline characteristics of frail older people with diabetes such as body weight, BMI, waist circumference, lipid profile, glycaemic control, hyperglycaemic or hypoglycaemic episodes, serum albumin, Hb, or other metabolic characteristics. The exclusion criteria included non-English language, studies conducted on patients without diabetes or frailty diagnosis, studies on younger (<55 years) age groups, review articles, editorials, abstracts, conference proceedings, non-human studies, or expert opinions. 

### 3.3. Data Extraction

We independently reviewed the studies and performed data extraction in a standardised format. For each study, data were extracted in 4 main categories: 1. Author, study design, year of publication, and country of origin. 2. Baseline data, which included the number of patients, mean age, and duration of follow-up. 3. Aim of the study. 4. Main findings, which included all the reported characteristics of older people with diabetes and the outcomes. We grouped the studies describing patients’ characteristics that suggest an anorexic malnourished phenotype in one table and those suggesting a sarcopenic obese phenotype in another table. Frail patients, who had lower body weight, lower BMI, waist circumference, or lower HbA1c compared to non-frail patients were grouped under the AM phenotype while frail patients with opposite criteria, in comparison to non-frail patients were grouped under the SO phenotype. Disagreements were resolved by consensus between authors. PRISMA checklist is available in Supplementary Materials. 

## 4. Results

After accounting for the exclusion criteria, described in Figure 1, twenty-five studies were included in this review. Studies describing patients with criteria suggesting a malnourished phenotype are summarised in Table 1 while those describing patients with criteria suggesting a sarcopenic obese phenotype are summarised in Table 2. 

### 4.1. Studies Suggesting AM Phenotype

Fifteen studies described the physical and metabolic characteristics of frail patients who tended to have low glycaemia, low body weight, or BMI, compared to non-frail patients suggesting an AM phenotype. Chao et al. in their first study, found that patients with an increased risk of hypoglycaemia have more comorbidities, and diabetes-related complications, are less obese, less prevalent hyperlipidaemia, and less use of hypolipidaemic medications [14]. In their second study, they reported that frail, compared to non-frail patients, were older, less obese, had more prevalent comorbidities, more diabetes-related complications, more frequent hypoglycaemic episodes, less prevalent hyperlipidaemia, less use of hypolipidaemic medications, more insulin and less oral hypoglycaemic agents use [15]. Malnourished patients, as described by Nguyen et al., were older, underweight, more frail, more exhausted, and more cognitively impaired, compared to well-nourished patients [16]. In addition, malnutrition risk is increased by frailty and cognitive impairment [16]. Yanagita et al. described frail patients to be older, have lower body weight, lower HbA1c, lower HDL cholesterol, lower serum albumin, lower systolic blood pressure, lower Hb, less use of hypolipidaemic agents and similar use of hypoglycaemic agents compared to non-frail patients [17]. Similarly, the frail cohort, described by McAlister et al., were older, had lower BMI, lower HbA1c, lower cholesterol, and more comorbidities compared to non-frail patients [18]. de Decker et al. demonstrated that patients with more frequent episodes of hypoglycaemia have lower body weight, more comorbidities, more dependency, more dementia prevalence, more insulin, and less oral hypoglycaemic use compared with those without hypoglycaemia [19]. Cacciatore et al. reported that severely frail, compared to non-frail patients, are significantly older, had lower BMI, lower waist circumference, more comorbidities, more disabilities, more cognitive impairment, and higher use of insulin and oral hypoglycaemic therapy [20]. Case series described by Abdelhafiz et al. showed that patients in whom hypoglycaemic therapy was successfully withdrawn have significant weight loss, low HbA1c, and prevalent comorbidities, especially dementia [21]. Sjoblom et al. successfully reduced and/or withdrew hypoglycaemic therapy in the intervention arm of a cohort of nursing home residents [22]. In the intervention, compared to non-intervention, patients had lower HbA1c, lower BMI, and more comorbidities [22]. The study by Morita et al. showed that disabled patients with HbA1c of 6.0% to be older, have more prevalent hypoalbuminaemia and less hyperlipidaemia compared to those with HbA1c ≥ 7.0% [23]. Adame Perez et al. demonstrated that frail patients have lower lean body mass, lower skeletal muscle mass index, and more comorbidities compared to non-frail counterparts [24]. Kitamura et al. reported that frail patients to be older, had lower BMI and lower albumin compared to non-frail patients [25]. Thorpe et al. found weight loss, iron deficiency anaemia, older age, and comorbidities to be common in patients with tight glycaemic control (HbA1c < 7.0%) [26]. Sussman et al. demonstrated that patients with HbA1c < 6.0% are older, have more comorbidities, more metastatic cancer, more dementia, and limited life expectancy compared with those with HbA1c ≥ 6.0% [27]. Similarly, Yotsapon et al. reported that patients with low HbA1c (<7.0%) are older and have severe comorbidities and dementia [28]. 

### 4.2. Studies Suggesting SO Phenotype

Ten studies described physical and metabolic characteristics of frail patients who tended to have high glycaemia, high body weight, or obesity, compared to non-frail patients suggesting a SO phenotype. Kong et al. described a cohort of frail patients who were older and have significantly higher HbA1c, BMI, and more comorbidities than non-frail patients [29]. They also have more prevalent depressive symptoms, malnutrition, low income, and low exercise activity [29]. Similarly, Ferri-Guerra et al. reported that frail patients were significantly older, had more comorbidities, had a long duration of diabetes, more use of hypoglycaemic agents, and significantly higher BMI and HbA1c compared to non-frail patients [30]. In addition, frail patients had significantly higher hospitalisation and mortality compared with non-frail patients [30]. Hyde et al. showed that subjects with HbA1c levels ≥ 6.5% have the greatest prevalence of frailty (70.3%) [31]. Frailty was more common in obese subjects and was associated with higher HbA1c levels. In binary logistic regression analysis, having an HbA1c level ≥ 6.5% was associated with being frail after adjustment for age, sex, and education. However, this association was attenuated after further adjustment for BMI [31]. Aguayo et al. similarly reported that frailly was associated with high BMI, high HbA1c, sedentary lifestyle, lower social class, and lower income [32]. García-Esquinas et al. demonstrated that the risk of frailty increases with increasing age, abdominal obesity, triglycerides, and HbA1c [33]. However, adjustment of these associated risk factors attenuated the risk of frailty [33]. Nguyen et al., in their retrospective analysis of the ADVANCE trial, described a frail cohort, which had higher body weight, higher waist circumference, higher systolic blood pressure, and higher HbA1c than the non-frail cohort [34]. In addition, the frail cohort had a higher prevalence of comorbidities, diabetes-related complications, and polypharmacy [34]. Bilgin et al. found that frail patients to be older, have more comorbidities, higher HbA1c, higher fasting blood glucose, lower albumin, and lower Hb compared to non-frail patients [35]. However, there was no difference in body weight, waist circumference, or BMI [35]. Lin et al., in their cross-sectional study, reported that frail patients had higher HbA1c (>8.0%), older age, and more frequent hyperglycaemia but similar BMI and lipid profile to non-frail patients [36]. Lipska et al., in their cross-sectional study, demonstrated that frail patients with complex health status are significantly older, have higher BMI, more comorbidities, and disabilities but similar HbA1c levels compared to more healthy patients [37]. Thein et al. reported that frail patients are older, have more comorbidities, longer duration of diabetes, more medication use, less weekly physical exercise, non-significantly higher BMI and waist circumference, and similar cholesterol levels compared to non-frail patients [38]. 

## 5. Discussion

The results of these studies suggest that frail patients are morphologically and metabolically different. In addition, frail patients can fit into at least two metabolically different phenotypes. Their net insulin resistance, total body weight, their muscle/fat ratio, and their most dominant muscle fibre type determine the metabolic phenotype of the individual. 

Skeletal muscles, which comprise about 50% of total body mass and are responsible for 80% of blood glucose uptake, contain mainly two main types of muscle fibres with different metabolic properties [39,40]. Type II fibres have less fat oxidative capacity, leading to higher lipid storage, which increases insulin resistance and predisposes to glucose intolerance compared to type I fibres [41]. With increasing age, type II muscle fibres atrophy accounts for the majority of muscle loss and this is accentuated further when frailty develops [42,43,44]. Insulin resistance of the individual will be affected by the overall body weight and body composition including visceral fat and differential loss of muscle fibre types. Therefore, insulin resistance decreases due to the increased loss of type II muscle fibres that is associated with significant body weight loss, which leads to the development of the AM phenotype of frailty. On the other hand, type II muscle fibre loss, which is associated with visceral fat deposition and weight gain, leads to the development of the SO phenotype of frailty. The above studies demonstrated that the metabolically heterogeneous nature of frailty spans across a spectrum ranging from the AM on one side to a SO on the other side [8]. 

In the AM phenotype studies, the first Chao et al. study showed that patients with hypoglycaemia had 2-fold higher mortality than those without hypoglycaemia, which could be a reflection of their unmeasured frail condition as the actual cause of mortality, rather than the hypoglycaemic events [14]. This was more clear in their second study, which demonstrated that weight loss, as a component of the FRAIL scale, was particularly associated with adverse outcomes, and increased healthcare utilisation [15]. Therefore, unmeasured frailty could be the underlying confounding factor, in studies, which found that low HbA1c is associated with an increased risk of mortality [45]. This direct relationship between frailty and mortality has been reported by Cacciatore et al. [20]. Additionally, frailty was associated with disability and care home needs as demonstrated by Morita et al. [23]. Nguyen et al. [16] highlighted the link between malnutrition, frailty, and cognitive impairment suggesting an increased risk of cognitive impairment in the AM type of frailty, which may lead to a vicious circle of deterioration and poor outcomes as previously demonstrated [46,47]. The reported AM phenotype in the studies by Yanagita et al. and McAlister et al. suggests a reverse metabolism in this frail phenotype where cardiovascular risk factors appear to have a protective effect [17,18]. We previously reported a metabolic shift and a U-shaped relationship between traditional cardiovascular risks factors such as cholesterol level or blood pressure and adverse outcomes in AM frail older people [48]. In the AM phenotype, due to malnutrition, frequent episodes of hypoglycaemia are expected [19] and, deintensification of hypoglycaemic agents is possible [21,22]. The normalisation of HbA1c can occur after the withdrawal of all hypoglycaemic medications, a state called frailty-induced burnt-out diabetes [49]. The AM frail older people had tight glycaemic control, due to a mismatch between their low body weight and hypoglycaemic drug burden, suggesting the need for deintensification [26,27,28]. The frail patients described by Adame Perez et al. [24] and Kitamura et al. [25] with more comorbidities, lower muscle mass, and lower albumin but similar lipid profile, HbA1c and BMI compared with non-frail patients, may represent an intermediate criterion in the spectrum of frailty between the AM and SO phenotypes. 

In the SO phenotype studies, in addition to the high HbA1c and high BMI, sociodemographic factors such as a history of alcohol drinking, low income, sedentary lifestyle, malnutrition, and depressive symptoms were associated with frailty [29]. Although malnutrition was prevalent, more than 50% of the cohort was overweight or obese [29]. Malnutrition could be related to poor mobility, swallowing difficulties, or insufficient protein intake [50]. Depression is common in diabetes and the risk increases further when obesity is also present [51]. In this SO phenotype, it appears that obesity, a sedentary lifestyle, and depression are associated with frailty [52]. In addition, there is evidence to suggest a reciprocal interaction between depression and frailty [52]. The study by Ferri-Guerra et al. showed a significant higher rate of hospitalisation and mortality due to cardiovascular and renal causes, which is likely common in the SO phenotype of frailty [30]. Hyde et al. suggested that obesity could be a confounding factor between high HbA1c and risk of frailty [31]. In other words, obesity may be a mediator in the relationship between glycaemic control and frailty, or a shared risk factor for SO frail phenotype and diabetes [31]. However, it has been recently reported that diabetes and obesity independently carry equal risks for the development of frailty [53]. Aguayo GA et al. confirmed the findings of previous studies that SO frail phenotype is associated with a sedentary lifestyle, lower socioeconomic class, and lower income [32]. García-Esquinas et al. suggested that diabetes-associated frailty is partly explained by unhealthy behaviours, obesity, and poor glucose control consistent with a SO phenotype of frailty [33]. The retrospective analysis of the ADVANCE study demonstrated a SO phenotype, which has a higher prevalence of cardiovascular disease and an increased risk of adverse outcomes as expected in this phenotype [34]. Bilgin et al. and Lin et al. described frail patients with high glycaemia but normal body weight, which may suggest intermediate metabolic criteria, closer in the frailty spectrum to the SO phenotype [35,36]. Lipska et al. described a SO phenotype frail cohort. Although they have demonstrated no difference in HbA1c in frail compared to healthy cohorts, they have suggested that this could be due to inappropriate over-treatment in the frail group [37]. The frail cohort described by Thein et al. had intermediate criteria of a sedentary lifestyle but similar BMI to non-frail subjects and this may be due to the increased prevalence of cognitive impairment in the frail cohort, which is likely to be associated with malnutrition and less weight gain [38]. 

**Figure 2:**
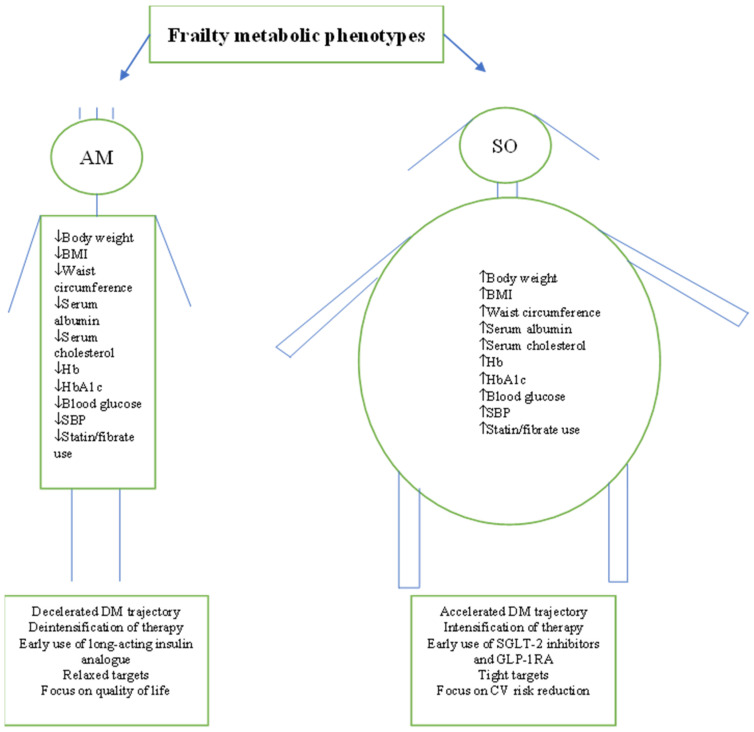
Summary of frail patients’ characteristics as reported by the studies. Characteristics suggest the existence of two distinctive metabolic phenotypes. AM = Anorexic malnourished, SO = Sarcopenic obese, BMI = Body mass index, Hb = Haemoglobin, SBP = Systolic blood pressure, DM = Diabetes mellitus, SGLT-2 = Sodium-glucose transporter, GLP-1RA = Glucagon-like peptide receptor agonists.

Although clinical guidelines divide older people dichotomously as frail and non-frail and consider frail people as one category, it appears from the above studies that frailty is a metabolically heterogenous condition, which will have important clinical implications. In the AM phenotype, the metabolism shifts and reverses leading to a decelerated course of diabetes trajectory [54]. On the other hand, the synergistic effect of sarcopenia and obesity in the SO phenotype accelerates the diabetes trajectory and accentuates the cardiovascular risk [54]. Therefore, the choice of hypoglycaemic agents and goals of therapy are different in each phenotype [55]. For example, in the AM phenotype, due to the significant weight loss and malnutrition, early use of insulin should be considered due to its potentially useful weight gain and anabolic properties [56,57,58,59,60]. Long-acting insulin analogues are the preferred choice compared with intermediate-acting human insulins due to their superior efficacy, a simple regimen of once-daily administration, and their overall safety including low risk of hypoglycaemia [61,62,63,64]. In addition, this phenotype is likely to need less intense hypoglycaemic therapy due to anorexia and weight loss. Therefore, deintensification of therapy, relaxed glycaemic targets of HbA1c (8.0–8.9%, 63.9–73.8 mmol/mol) adequate nutrition, and resistance exercise training are appropriate in this phenotype with a main goal of therapy to maintain the quality of life [65,66,67]. The combination of sarcopenia and obesity in the SO phenotype is associated with an unfavourable metabolic state that increases the risk of cardiovascular events and mortality compared to sarcopenia or obesity alone [68,69,70,71]. Therefore in this phenotype, the early use of sodium-glucose transporter-2 (SGLT-2) inhibitors and the glucagon-like peptide-1 receptor agonists (GLP-1RA) should be considered due to their cardiorenal protective effect, weight reduction properties, and their favourable effect on the metabolism [72,73,74,75,76,77,78,79,80,81]. Due to the high cardiovascular risk in this phenotype, intensification of therapy should be considered. The benefit of SGLT-2 inhibitors and GLP-1RA is independent of glycaemic control but target HbA1c < 7.0%, (53 mmol/mol) may be associated with better physical function [65]. Therefore, in this phenotype, intensification of hypoglycaemic therapy, prevention of cardiovascular risk factors, exercise training, and achieving ideal body weight to reduce cardiovascular events should be the main goal of therapy [82,83]. Figure 2. In addition to hypoglycaemic therapy, adequate nutrition combined with exercise training are the best strategy for improving metabolic profile, preservation of lean muscle mass, and achieving ideal body weight. Serum albumin levels are associated with inflammation and inversely related to frailty; therefore, in the AM phenotype with low albumin, a higher daily protein intake of 1–1.2 g/kg is required [84,85]. In the SO phenotype, weight loss via energy restriction alone, without exercise, should be avoided because it may cause simultaneous loss of muscle mass. Structured multimodal intervention, which included nutrition, resistance exercise training, and optimal diabetes care led to a clinically relevant and cost-effective improvement in the functional status of older frail and pre-frail participants with type 2 diabetes mellitus [67]. 

The strength of this review is that it highlights a new concept that frailty is a metabolically heterogeneous condition, which will have a significant impact on clinicians’ choice of hypoglycaemic agents and deciding the overall goal of therapy. It also helps to clarifyto clarify why both hypoglycaemia and hyperglycaemia are associated with frailty. Hypoglycaemia is likely associated with the AM phenotype, while hyperglycaemia is associated with the SO phenotype, explaining this apparent contradiction [86]. This manuscript is limited by the nature of the studies included in the review, as none of these studies was designed, at the outset, to address the question of metabolic phenotypes therefore, the metabolic and/or anthropometric data was not fully reported. However, the available data obtained from these studies appears to provide reasonable evidence that frailty is not a homogenous metabolic condition. In addition, we were not able to characterise the patients who have intermediate characteristics between the two main phenotypes, although this will need future studies specifically designed to address this question. As another limitation, we included studies of relatively younger patients (age ≥55 years) to make sure we include as many studies as possible to support our hypothesis. However, this could be also an advantage as although frailty prevalence increases with age, frailty can affect younger people. 

## 6. Conclusions

Current literature suggests that frailty in older people with diabetes is a metabolically heterogeneous condition. It is likely to span a metabolic spectrum that starts with an anorexic malnourished phenotype at one end to a sarcopenic obese phenotype at the other end. Therefore, clinical decision-making about hypoglycaemic agents’ choice, glycaemic goals, and future clinical studies should consider the metabolic heterogeneity of frailty. 

## 7. Future Perspectives

The search for metabolic phenotypes of frailty is a new concept that may help clinicians to practice more precise medicine. Different subtypes of type 2 diabetes have been previously reported based on different patient characteristics, insulin resistance, disease progression, and the risk of diabetes complications, which may help to accurately tailor clinical interventions [87]. Therefore, in future clinical studies, frail older people need to be characterised, not only based on their age and physical function but also on their metabolic characteristics to clearly identify who benefits most from which drug therapy and understand their diabetes trajectory. In addition, further research is still required to explore the differences in insulin sensitivity in skeletal muscle fibres, the age and frailty-related differential muscle fibre loss, and its impact on the overall insulin resistance of the individual. Interesting observations are the association of the SO phenotype with accelerated cardiovascular risk due to the synergistic action between sarcopenia and obesity, which significantly increases insulin resistance and accelerates the occurrence of cardiovascular events. The other observation is the association of the AM phenotype with dementia. This has been referred to previously as a reciprocal relationship between dementia, frailty, and hypoglycaemia, which leads to a downhill spiral of deterioration leading to the AM phenotype [88]. We were not able to explore these areas further as it is beyond the scope of this manuscript and is likely to need future research, which will help characterise frailty phenotypes further from a prognostic point of view. The newly developed hypoglycaemic agents of SGLT-2 inhibitors and GLP-1RA have unique anti-diabetes effects and prognostic benefits that are largely based on their favourable metabolic properties. The future investigations of hypoglycaemic therapy are likely to follow this direction, therefore identification of the metabolic phenotypes of frailty in future drug studies is important. Furthermore, drug studies are largely based on cardiovascular events, as the main outcome and the effect of novel drugs on outcomes more relevant for older people such as frailty and muscle function are still required. 

## 8. Key Points

- In older people, frailty is an important diabetes-related complication;
- Frailty is not a metabolically homogeneous condition;
- Current literature suggests that frailty is a spectrum that spans from an anorexic malnourished to sarcopenic obese phenotypes;
- The anorexic malnourished phenotype is associated with less insulin resistance and a high risk of hypoglycaemia while the sarcopenic obese phenotype is characterised by high insulin resistance and less risk of hypoglycaemia;
- Future studies are still required to further characterise the metabolic spectrum of frailty.

## Figures and Tables

**Table 1:** Studies suggesting AM frail phenotype.

Study	Patients	Aim to	Main Findings
Chao CT, et al., prospective, Taiwan, 2020 [14]	2119 patients with and 8432 without hypoglycaemia (control), mean (SD) age 65.9 (14) Y.	Examine whether hypoglycaemia increases the risk of frailty.	Patients with compared to those without hypoglycaemia had: A. Less obesity (1.4% vs. 2.4%, *p* = 0.006). B. Less hyperlipidaemia (55.5% vs. 63.0%, *p* < 0.001). C. Higher mean (SD) CCIS, 4.2 (2.4) vs. 3.4 (2.2), *p* < 0.001. D. Higher mean (SD) aDCSI, 1.3 (1.5) vs. 0.8 (1.2), *p* < 0.001. D. Less patients using statin (36.4% vs. 44.8%, *p* < 0.001) or fibrate (12.4% vs. 16.1%, *p* < 0.001). E. No difference in hypoglycaemic therapy use.
Chao CT, et al., prospective, Taiwan, 2018 [15]	560,795 patients with DM, mean (SD) age 56.4 (13.8) Y.	Examine the association of pre-frailty and frailty with mortality.	Frail (≥3 components in FRAIL scale) compared to non-frail patients had: A. Higher mean (SD) age, 75.1 (11.2) vs. 54.8 (13.2), *p* < 0.01. B. Higher mean (SD) aDCSI, 1.4 (1.5) vs. 0.2 (0.6), *p* < 0.01. C. Higher mean (SD) CCIS, 5.4 (2.4) vs. 1.7 (1.4), *p* < 0.01. D. More prevalence of hypertension, CKD, CLD, COPD, AF, CAD, PVD, gout, OA, malignancy, osteoporosis, mental illness, and CVD, all *p* < 0.01. E. Less hyperlipidaemia, 31.9% vs. 41.2%, *p* < 0.01. F. Less obesity, 0.9% vs. 1.6%, *p* < 0.01. G. More hypoglycaemia, 1.2% vs. 0.1%, *p* < 0.01. H. Less statin and fibrate use, *p* < 0.01. I. More insulin users, 7.7% vs. 4.8%, *p* < 0.01. J. Less oral hypoglycaemic use, *p* < 0.01.
Nguyen TTH, et al., cross-sectional, Vietnam, 2019 [16]	158 subjects with DM, mean (SD) age 69.52 (6.76) Y.	Assess nutritional status using the Mini-Nutrition Assessment Short Form tool, and describe the relationship among related factors.	Malnourished compared to well-nourished had: A. Older (≥80 Y) subjects, 11.5% vs. 6.6%, *p* = 0.27. B. More underweight (10.2% vs. 0.0%) and less overweight subjects (30.6 vs. 71.6), *p* < 0.001. C. More exhausted subjects, 30.8% vs. 11.0%, *p* < 0.01. D. More cognitive impairment, 53.1% vs. 27.5%, *p* = 0.002. E. More frail subjects, 20.4% vs. 2.8%, *p* < 0.001. F. Malnutrition risk increased by frailty (OR 9.06, 95% CI 2.37 to 34.65, *p* = 0.001) and cognitive impairment, 2.98, 1.48 to 6.0, *p* = 0.002.
Yanagita I, et al., cross-sectional, prospective, Japan, 2018 [17]	132 hospitalised patients with DM, mean (SD) age 78.3 (8.0) Y.	Evaluate frailty risk factors including HbA1c using CFS.	Frail compared to non-frail had: A. Higher mean (SD) age, 82.78 (8.16) vs. 75.17 (6.20) Y, *p* < 0.001. B. Lower mean (SD) HbA1c, 6.6 (0.93) vs. 7.27 (1.04)%, *p* < 0.001). B. Lower mean (SD) albumin, 35.62 (4.82) vs. 41.73 g/L, *p* < 0.001). C. Lower mean (SD) HDL-cholesterol, 1.23 (0.39) vs. 1.46 9 (0.43) mmol/L, *p* < 0.01). D. Lower mean (SD) Hb, 117.89 (17.28) vs. 132.38 (17.02) g/L, *p* < 0.001. E. Lower mean (SD) SBP (KPa) 16.81 (2.12) vs. 17.60 (1.92), *p* < 0.05). F. Lower mean (SD) body weight, 51.64 (12.72) vs. 59.17 (10.94) Kg, *p* < 0.001). G. Fewer patients using statin or fibrate (%) 34.54 vs. 54.54, *p* < 0.05. H. No difference in hypoglycaemic therapy use.
McAlister FA, et al., retrospective, UK, 2018 [18]	292,170 subjects with DM, mean (SD) age 61.7 (15.6) Y.	Examine glycaemic control across health states.	Frail compared to non-frail subjects had: A. Higher mean (SD) age, 72.2 (11.2) vs. 60.8 (15.5), *p* < 0.0001. B. Higher mean (SD) CCIS, 2.73 (1.6) vs. 1.04 (0.86), *p* < 0.0001. C. Lower mean (SD) BMI, 26.0 (6.8) vs. 30.17 (6.9), *p* < 0.0001. D. Lower mean (SD) HbA1c, 7.1 (1.46) vs. 7.38 (1.64)%, *p* < 0.0001. E. Lower total cholesterol (*p* = 0.007), triglycerides (*p* = 0.05) and LDL-cholesterol (0.001). F. More comorbidities and diabetes complications, *p* < 0.0001.
De Decker L, et al., cross-sectional, France, 2017 [19]	1552 patients with DM, mean (SD) age 86.4 (4.4) Y.	Determine the association between hypoglycaemia and a high burden of comorbidities.	Patients with compared to those without hypoglycaemia had the mean (SD): A. Lower body weight (Kg) 65.80 (14.7) vs. 69.5 (24.5), *p* = 0.004). B. Lower eGFR (ml/min) 46.1 (20.8) vs. 51.9 (26.1), *p* < 0.001. C. Higher CCIS 4.7 (2.3) vs. 3.8 (2.1), *p* < 0.001. C. Greater level of dependency (*p* < 0.001). D. Higher prevalence of CV disease (%) 68.6% vs. 54.3%, *p* < 0.001. E. Higher prevalence of dementia (%) 67% vs. 59.3%, *p* < 0.006. F. More use of insulin, *p* < 0.001 G. More SMBG (%) 84.6% vs. 67.8, *p* < 0.001. H. Lower use of SU, metformin, *p* < 0.001 and glinides, *p* = 0.002.
Cacciatore F, et al., prospective, Italy, 2013 [20]	1288 subjects, mean (SD) 72.4 (6.3) Y, F/U 12 Y.	Examine the predictive role of frailty on long-term mortality.	Severely frail compared to non-frail patients had: A. Higher mean (SD) age, 78.9 (6.0) vs. 72.1 (4.7), *p* < 0.001. B. Lower mean (SD) BMI, 26.4 (5.1) vs. 27.0 (3.6), *p* = 0.087. C. Lower mean (SD) waist circumference, 94.8 (19.2) vs. 98.8 (14.9), *p* = 0.672. D. Higher mean (SD) CCIS, 4.8 (2.2) vs. 2.2 (1.6), *p* < 0.001. E. More insulin users, 26.0% vs. 13.4%, *p* = 0.004. F. More hypoglycaemic drug users 87.2% vs. 73.1, *p* = 0.008. G. More comorbidities of CHF, CKD, low MMSE, high GDS, disability, and mortality, all *p* < 0.001.
Abdelhafiz AH, case series, UK, 2014 [21]	8.0 patients with type 2 DM, mean (SD) age 86.5 (3.2) Y and tight glycaemic control.	Describe whether hypoglycaemic therapy can be withdrawn in patients with HbA1c ≤6.0% or having recurrent hypoglycaemia.	Mean (SD) HbA1c was: A. 6.2% (0.8) at the point of medication withdrawal. B. 6.5% (0.7) after one year of follow-up, *p* = 0.4. At the point of medication withdrawal compared to the initial point of starting medication: A. Increased mean number of comorbidities 6.8 vs. 4.1, *p* = 0.002. B. Decreased mean weight 75.4 vs. 88.0 Kg, *p* = 0.003. C. Increased mean number of medications 10.1 vs. 6.4, *p* = 0.01. D. 50.0% of patients had a new diagnosis of dementia.
Sjoblom P, et al., observational, Sweden, 2008 [22]	32.0 nursing home patients with DM, mean (SD) age 84.4 (6.8) Y.	Explore the feasibility of hypoglycaemic medication withdrawal in patients with HbA1c ≤6.0%.	Intervention compared to the non-intervention group had: A. Lower mean (SD) BMI, 25.6 (4.5) vs. 26.5 (5.1). B. Lower eGFR 50 vs. 55 mL/min/1.73 m^2^. C. Longer mean (SD) duration of DM, 10.6 (8.9) vs. 9.0 (7.4) Y. D. Lower mean (SD) HbA1c 5.2 (0.4) vs. 7.1 (1.6).
Morita T, et al., prospective, Japan, 2017 [23]	184 patients with diabetes aged 65–94 Y, F/U 5 Y.	Investigate if low HbA1c is associated with risk of support/care need certification.	42 (22.8%) patients developed disability defined as a requirement of first support/care-need certification. Compared to patients with HbA1c ≥ 7.0%, patients with HbA1c < 0.6% were: A. Older, mean (SD) age 77.5 (6.5) vs. 75.1 (6.3) Y, and had more people aged >75 Y, 68.5% vs. 54.5%, Kruskal–Wallis *p* < 0.05/3 and 0.20/3 respectively. B. Had less insulin use 37.8% vs. 63.6%, *p* < 0.20/3. C. Had less dyslipidaemia 37.0% vs. 59.1%, *p* < 0.05/3. D. Had more hypoalbuminaemia 14.8% vs. 6.8%, *p* < 0.20/3.
Adame Perez SI, et al., cross-sectional, Canada, 2019 [24]	41 subjects with DM, median (IQR) age 70.0 (65.0–74.0) Y.	Compare differences in body composition by frailty status.	Frail compared to non-frail patients had: A. Lower mean (SD) ASMI (kg/m^2^), 6.8 (1.0) vs. 7.7 (0.9), *p* = 0.02. B. More patients with low lean body mass, 57.1% vs. 14.7%, *p* = 0.01. C. Higher mean (SD) comorbidities, 6.0 (2.0) vs. 4.0 (2.0), *p* = 0.03. C. No difference in body weight, BMI, HbA1c, hypoglycaemic therapies, or hypoglycaemic episodes.
Kitamura A, et al., prospective, Japan, 2019 [25]	1271 subjects, mean (SD) age 71.0 (5.6) Y, 176 had DM.	Clarify risks of death and disability.	A. Frail compared to non-frail were older 72.5 vs. 69.6 Y, *p* = 0.05, more patients had hypoalbuminaemia, 20.7 vs. 5.9%, *p* = 0.02 and lower BMI 20.8 vs. 2.0%, *p* = 0.06. B. There was no difference in HbA1c, lipid profile, average BMI, hypoglycaemic medications, and comorbidities.
Thorpe CT, retrospective, US, 2015 [26]	15,880 patients ≥ 65.0 years old with DM and dementia.	Examine: A. Risk factors for tight glycaemic control B. Medications associated with the risk of hypoglycaemia.	A. 52.0% of patients had tight glycaemic control (HbA1c < 7.0%). B. Factors associated with tight control were: 1. Older age (75.0–84.0 years, OR 1.16, 95.0% CI 1.07 to 1.126, *p* = 0.001, ≥75.0 years, (1.13, 1.02 to 1.125, *p* = 0.021. 2. Heart valve disease (OR 1.16, 95.0% CI 1.01 to 1.32, *p* = 0.033), chronic lung disease OR 1.10, 95.0% CI 1.01 to 1.21, *p* = 0.038), and deficiency anaemia (OR 1.12, 95.0% CI 1.02 to 1.22, *p* = 0.016). 3. Weight loss (OR 1.36, 95.0% CI 1.09 to 1.69, *p* = 0.006). C. Among tightly controlled patients, 75.0% used SU and/or insulin.
Sussman JB, et al., retrospective, US, 2015 [27]	179,991 patients > 70.0 years old on active treatment for DM.	Examine the rate of medications deintensification.	Patients with very low HbA1c (<6.0%) compared to those with higher HbA1c (≥6.5%) were: A. Older (mean age 78.6 vs. 77.8 years). B. More comorbidities (mean CCIS 1.44 vs. 1.27). C. More low life expectancy <5.0 years (19.9% vs. 15.7%). D. More dementia (2.3 vs. 1.6%). E. More palliation in prior year (0.5 vs. 0.3%). F. More metastatic cancer (0.7 vs. 0.4%).
Yotsapont, et al., retrospective, Thailand, 2015 [28]	143.0 patients > 85.0 years old with DM.	Describe clinical characteristics and outcomes of “oldest old” patients with DM.	Patients had: 1. Long duration of diabetes, mean (SD) 22.1 (11.1) Y. 2. Severe comorbidities, CCIS ≥ 5.0 in 35.3%. 3. Tight glycaemic control, HbA1c < 7.0% in 66.9%. 4. Frequent hypoglycaemia in 10.5%. 5. Multiple comorbidities: 23.4% diabetic retinopathy, 54.9% CKD, 15.8% CV disease, 18.0% stroke, 22.6% dementia. 6. Only 20.0% of those with HbA1c <6.0% received medication deintensification.

SD = Standard deviation, Y = Year, CCIS = Charlson Comorbidity Index Score, aDCSI = Adjusted diabetic complication severity index, DM = Diabetes mellitus, CKD = Chronic kidney disease, CLD = Chronic liver disease, COPD = Chronic obstructive pulmonary disease, AF = Atrial fibrillation, CAD = Coronary artery disease, PVD = Peripheral vascular disease, OA = Osteoarthritis, CVD = Cerebrovascular disease, OR = Odds ratio, CI = Confidence interval, CFS = Clinical frailty scale, Hb = Haemoglobin, SBP = Systolic blood pressure, BMI = Body mass index, eGFR = Estimated glomerular filtration rate, CV = Cardiovascular, SMBG = Self-monitoring blood glucose, SU = Sulfonyl urea, CHF = Congestive heart failure, MMSE = Mini-mental state examination, GDS = Geriatric depression scale, F/U = Follow up, ASMI= Appendicular skeletal muscle mass index, OR = Odds ratio.

**Table 2:** Studies suggesting SO frail phenotype.

Study	Patients	Aim to	Main Findings
Kong L, et al., cross-sectional, China, 2021 [29]	291 community-dwelling older people, median (IQR) age 69 Y (IQR 67–72) with DM.	Identify predictors of frailty.	Frail compared with non-frail patients were: A. Older (% ≥ 75 Y) 19.6% vs. 9.4%. B. Significantly higher HbA1c, median (IQR) 6.97% (5.95, 8.42) vs. 6.74% (5.96, 7.20), *p* = 0.055. C. Significant comorbidities, median (IQR) 5.0 (4,7) vs. 4.0 (3,6), *p* = 0.030. D. Higher BMI (% ≥ 28) 17.9% vs. 11.8%.
Ferri-Guerra J, et al., retrospective, US, 2020 [30]	763 patients with DM, mean (SD) age 72.9 (6.8) Y.	Determine the association of frailty with all-cause hospitalisations and mortality.	Frail compared to non-frail patients had: A. Higher mean (SD) age, 73.33 (7.26) vs. 72.4 (6.23), *p* = 0.19. B. Higher mean (SD) BMI, 30.07 (6.0) vs. 29.82(5.15), *p* = 0.54. C. More end-organ damage, 36.4% vs. 28.3%, *p* = 0.02 D. Higher mean (SD) DM duration, 9.48 (5.21) vs. 8.46 (5.36), *p* < 0.009. E. Less patients with HbA1c ≤ 7 and more patients with HbA1c > 7%, *p* = 0.15. F. Higher mean (SD) CCIS, 6.91 (2.0) vs. 5.75 (1.65), *p* < 0.0001. F. More hypoglycaemic drug users 87.2% vs. 73.1, *p* = 0.008. G. More polypharmacy, hospitalisation and mortality, all *p* < 0.0001.
Hyde Z, et al., cross-sectional, Australia, 2019 [31]	141 Aboriginal Australian, mean (SD) age 62.2 (11.1) Y.	Explore whether HbA1c is associated with frailty.	A. Mean (SD) BMI of participants 28.5 (7.1) kg/m^2^ (range 13.9–52.1). B. 31.2% obese (BMI ≥ 30.0), 29.1% overweight (25.0–29.9), 19.2 normal (20.0–24.9), and 8.5 underweight (≤19.9). C. Association between HbA1c ≥ 6.5% and frailty was attenuated after adjustment for BMI (OR 2.10, 95% CI 0.92 to 4.80). D. Frailty is more common in obese subjects (70.5%) but similar in other groups, 50.0% in underweight, 59.3% in normal weight, and 53.7% in overweight. E. BMI was a possible confounder in the association between HbA1c and frailty.
Aguayo GA, et al., prospective, UK, 2019 [32]	5377 participants, median (IQR) 70 (65, 77 Y, F/U 10 Y.	Examine whether individuals with DM or high HbA1c experience different frailty trajectories with ageing.	Patients with compared to those without DM had, mean (SD): A. HbA1c 7.0% (0.4) vs. 5.5% (0.5). B. BMI 30.1 (4.8) vs. 27.4 (4.8). C. More obesity 45% vs. 26%. D. Lower income 27% vs. 35%. E. Less high social class 29% vs. 34%. F. Less high physical activity 51% vs. 67%. G. Frailty index (%frailty) 53% vs. 32%. H. EFS (%frail) 19% vs. 10%. I. Phenotype of frailty (%frail) 23% vs. 13%
García-Esquinas E, et al., prospective, Spain, 2015 [33]	1750 subjects aged ≥60 Y, 346 with DM, F/U 3.5 Y.	Assess the risk of incident frailty.	115 Cases of incident frailty were ascertained, baseline variables and risk of frailty were: age (OR 1.13, 95% CI 1.10 to 1.16), abdominal obesity (2.64, 1.61 to 4.33), triglycerides (1.04, 1.02 to 1.05) and HbA1c 1.48, 1.20 to 1.81).
Nguyen TN et al., retrospective, multicentre, 2021 [34]	Total 11,140 subjects with DM, mean (SD) age 65.78 (6.39) Y.	Develop a FI and explore the relationship of frailty to subsequent adverse outcomes.	Frail compared with non-frail patients had: A. Higher mean (SD) age 66.27 (6.79) vs. 65.60 (6.24). B. Higher mean (SD) SBP 153.47 (23.72) vs. 142.09 (19.91). C. More overweight, 86.1% vs. 68.0%. D. Higher mean (SD) waist circumference, 104.40 (13.05) vs. 96.49 (12.51). E. Higher mean (SD) HbA1c, 7.85 (1.70) vs. 7.40 (1.48). F. More comorbidities, DM-related complications, and polypharmacy.
Bilgin S, et al., cross-sectional, Turkey, 2020 [35]	101 patients with DM (41 frail, 60 not frail). Mean (SD) age 64.2 (8.0) Y frail, 62.2 (7.0) Y non-frail.	Observe clinical and laboratory indices of frail and non-frail patients with DM using Edmonton frail score.	Frail compared with non-frail patients: A. Median (IQR) Edmonton frail score 9.0 (7, 13) frail and 4.0 (1, 6) non-frail, *p* < 0.001. B. 71% frail and 48% non-frail had poorly controlled DM, *p* = 0.03. C. Fasting blood glucose (*p* = 0.02), HDL cholesterol (*p* = 0.005), and HbA1c (*p* = 0.04) were significantly higher in frail compared to non-frail. D. Serum triglyceride (*p* = 0.04), serum albumin (*p* = 0.006), Hb (*p* = 0.04), and eGFR (*p* = 0.01) were significantly lower in frail compared to non-frail. E. BMI, body weight, waist circumference, LDL-cholesterol, and total cholesterol were not significantly different between both groups.
Lin CL, et al., cross-sectional, Taiwan, 2022 [36]	248 subjects with DM, mean (SD) age 73.9 (5.9) Y.	Estimate prevalence and investigate risk factors of frailty.	Frail compared to non-frail had: A. Higher mean (SD) age 75.9 (5.7) vs. 73.1 (5.8), *p* = 0.001. B. More patients with HbA1c >8.0%, 22.7%vs 12.1%, *p* = 0.04. C. Similar BMI and lipid profile. D. Higher frequency of hyperglycaemic episodes, *p* = 0.001. E. More ADL disability, cognitive impairment, and depression, *p* < 0.001.
Lipska KJ, et al., cross-sectional, US, 2015 [37]	1288 non-institutionalised older people ≥ 65.0 years with DM.	Explore the prevalence of overtreatment of DM by health status.	Poor health status compared to healthy patients had: A. Higher mean (SD) age, 74.9 (6.0) vs. 72.0 (5.2) Y. B. Higher mean (SD) BMI, 32.6 (8.4) vs. 30.0 (5.6). C. More mean (SD) comorbidities, 2.9 (1.4) vs. 1.2 (0.7). D. More impairment in ≥1 ADL, 98.5% vs. 13.1%. E. More impairment in ≥1 IADL, 81.7% vs. 17.1%. F. No differences in HbA1c levels.
Thein FS, et al., prospective, Singapore, 2018 [38]	486 subjects with DM, mean (SD) age 67.3 (7.5) Y.	Investigate the prevalence of cognitive impairment and/or physical frailty.	Frail compared to non-frail subjects had: A. Higher mean (SD) age 76.9 (8.0) vs. 66.0 (6.7), *p* < 0.0001. B. Longer mean (SD) duration of DM, 13.7 (8.8) vs. 8.6 (7.5), *p* = 0.04. C. More mean (SD) number of morbidities, 1.9 (1.5) vs. 1.4 (1.1), *p* = 0.01. D. More patients with polypharmacy, 47.8% vs. 28.6%, *p* = 0.04. E. Less physical exercise per week, *p* = 0.014. F. Similar mean (SD) BMI, 25.1 (8.7) vs. 24.9 (3.7), *p* = 0.9. G. Similar mean (SD) waist circumference, 86.9 (14.0) vs. 86.5 (9.4), *p* = 0.84. H. Similar mean (SD) total cholesterol, 5.1 (1.02) vs. 5.0 (0.97), *p* = 0.13.

IQR = Inter quartile range, Y = Year, DM = Diabetes mellitus, FI = Frailty index, BMI = Body mass index, SD = Standard deviation, CCIS = Charlson Comorbidity Index Score, OR = Odds ratio, CI = Confidence interval, F/U = Follow up, EFS = Electronic frailty scale, FI = Frailty index, SBP = Systolic blood pressure, ADL = Activities of daily living, IADL = Instrumental activities of daily living.

## Supplementary Materials

The following supporting information can be downloaded at: https://www.mdpi.com/article/10.3390/metabo13060705/s1.
